# Age as a Predictive Factor in Severity of Injuries in Riders of Electric Bikes and Powered Scooters: A Retrospective Cross-Sectional Study

**DOI:** 10.3390/healthcare10091689

**Published:** 2022-09-04

**Authors:** Yafit Hamzani, Helena Demtriou, Adi Zelnik, Nir Cohen, Michael J. Drescher, Gavriel Chaushu, Bahaa Haj Yahya

**Affiliations:** 1 Department of Oral and Maxillofacial Surgery, Rabin Medical Center—Beilinson Hospital, Petach Tikva 4941492, Israel; 2 Maccabi-Dent, Holon 5810001, Israel; 3 Department of Orthopedic Surgery, Rabin Medical Center—Beilinson Hospital, Petach Tikva 4941492, Israel; 4 Sackler Faculty of Medicine, Tel Aviv University, Tel Aviv 6997801, Israel; 5 Department of Emergency Medicine, Rabin Medical Center—Beilinson Hospital, Petach Tikva 4941492, Israel; 6 The Maurice and Gabriela Goldschleger School of Dental Medicine, Tel Aviv University, Tel Aviv 6997801, Israel; 7 Oral and Maxillofacial Private Clinic, Herzliya 4685107, Israel

**Keywords:** emergency department, age, electric bikes, powered scooters, injury

## Abstract

The growth in worldwide popularity of electric bikes (E-bikes) and powered scooters (P-scooters) has been accompanied by an increase in injuries associated with their use. The aim of this study was to evaluate the contribution of rider age to injury severity, represented by need for hospitalization. A retrospective review of the database of a tertiary medical center yielded 1234 patients (75.7% male) who attended the emergency department (ED) in 2014–2020 for injuries sustained while riding an E-bike or P-scooter. Mean age was 31.52 ± 14.77 years: 23% were aged <20 years; 33%, 21–30 years; 23%, 31–40 years; 10%, 41–50 years; 11%, >51 years. Ninety patients (7.3%) were hospitalized. Older age was significantly associated with the need for hospitalization on univariate analysis (*p* <.001), but significance was not maintained on binary logistic regression (OR = 1.02, 95%CI 0.99–1.06; *p* = 0.11). Patients who underwent imaging evaluation in the ED were at lower risk of hospitalization, and patients who had surgery or a relatively long operative procedure were at higher risk of hospitalization. The study shows that older age (>51 years) is not associated with a significantly increased probability of severe injury in E-bike and P-scooter riders. This finding has important implications for insurers and healthcare administrators.

## 1. Introduction

The increasing worldwide popularity of electric bicycles (E-bikes) and powered scooters (P-scooters) has been accompanied by an increase in injuries associated with their use [1,2,3]. Most of the injuries are categorized as high-energy trauma and mainly affect the head and upper extremities [4]. The injured riders involved are usually healthy males in their 30s [1,2,3,4,5].

Recent studies have investigated potential factors that may contribute to the incidence and severity of injuries involving E-bikes and P-scooters, such as helmet use and alcohol consumption [1,2,3,5,6,7,8,9,10,11]. Rider age warrants particular attention in this context given the spiraling growth of the aging population and its impact on economic growth, political decision making, social needs, and healthcare management [12,13]. Between 2007 and 2017, the number of adults aged 60 years and over in the United States rose from 52 million to 71 million [14]. During this period, the rate of emergency department (ED) visits increased proportionally, along with an increase in the percentage of visits in which patients arrived by ambulance and in which patients were referred for hospitalization from the ED [14].

It is recognized that advanced age and comorbidities have a crucial impact on the probability of requiring extensive medical care in cases of injuries [12]. The aim of the present study was to determine if age is a contributory factor to the severity of injuries sustained by riders of E-bikes and P-scooters. The study hypothesis was that older age will result in an increase in incidence and severity of those electric vehicle injuries.

## 2. Materials and Methods

A retrospective, cross-sectional study was performed in the ED of a tertiary medical center in Israel from January 2014 to March 2020. A primary search of the healthcare database was conducted using the keywords “electric scooter” or/and “electric bike” and/or “powered scooter” or/and “powered bike” and “injury/injured”. Of the 1417 patients identified, 1234 were actually involved in an E-bike or P-scooter accident and had sufficient available data for inclusion in the study. The following parameters were collected from the medical files: demographics (age, gender), type of two-wheel electric vehicle used, hospitalization (yes/no), length of hospitalization (if relevant), use of imaging, type of imaging (if relevant), surgery (yes/no), duration of surgery (if relevant), and status at the end of the ED visit. Findings were compared between patients who required hospitalization and those who did not. The study protocol was approved by the Helsinki Committee of Rabin Medical Center (approval number 0194-20-RMC).

The data were analyzed with SPSS statistical software, version 25 (IBM^®^, Armonk, NY, USA). Continuous variables were summarized by mean and standard deviation, and discrete variables by frequency. Univariate analysis was performed using chi-square (χ2) test, and independent samples were analyzed with Mann–Whitney test. Significance was set at a *p*-value lower than 5%.

## 3. Results

### 3.1. Patient Demographics

**Figure 1:**
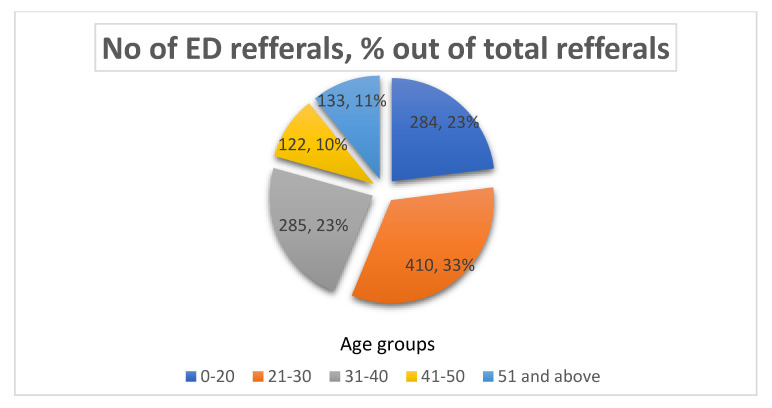
Pie distribution of ED referrals by age groups.

The demographic and clinical characteristics of the patients are shown in Table 1. The cohort was comprised of 934 men (75.7%) and 300 women (24.3%). Mean age was 31.52 ± 14.77 years and median age was 28 years. A total of 284 patients (23.0%) were aged <20 years; 410 (33.2%), 21–30 years; 285 (23.1%), 31–40 years; 122 (9.9%), 41–50 years; and 133 (10.8%, >51 years, as seen in Figure 1. Most of the accidents (79.5%) involved E-bikes.

**Figure 2:**
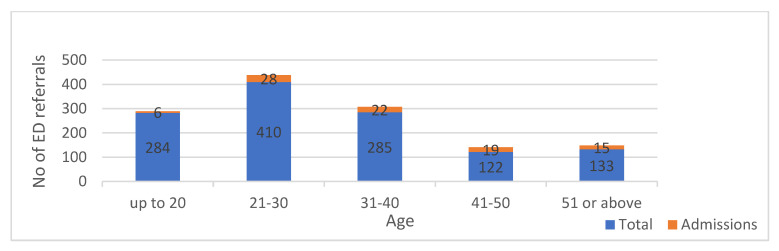
Number of admissions from total cohort by age groups.

**Figure 3:**
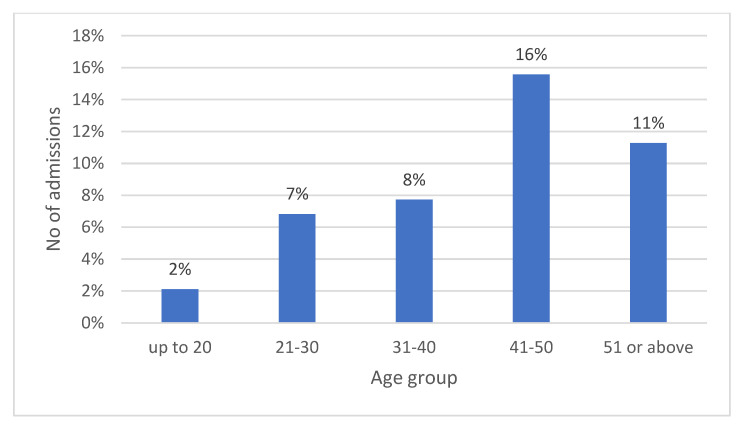
Percentage of admissions from total cohort by age groups.

Ninety patients in the cohort (7.3%) required hospitalization. Most (56%) were in their third or fourth decade; 28 patients were aged 21–30 years; and 22 were aged 31–40 years. Although individuals aged 41–50 years and 51+ years accounted for the lowest proportion of patients who visited the ED (21% of the cohort), they had the highest admission rates, of 16.4% (20/122) and 11.3% (15/133), respectively. Figure 2 and Figure 3 show the number and percentage of hospitalized patients by age group. The mean number of hospital admission days was 5.44 ± 0.12.

Imaging technologies were used as part of the ED work-up in 1020 patients (82.7%). They mainly included plane radiographs, in 83.2% of the cohort. Surgery was required in 212 patients (17.2%). At the end of the ED visit, 98.8% of the cohort was discharged home.

### 3.2. Relationship of Age and Other Variables with Hospitalization

As seen in Table 2, the continuous variables did not distribute normally, so nonparametric statistical methods were used. The results are shown in Table 3. On univariate analysis of the independent variables, a significant association was found between older age and the probability of hospitalization (*p* < 0.001). Mean age was 37.21 ± 14.34 years in the patients who were hospitalized compared to 31.08 ± 14.72 years in the patients who were not.

There was a statistically significant relationship between the absence of imaging technology during the ED visit work-up and hospitalization [χ^2^ (1) = 111.45, *p* < 0.001], with 57.8% of the hospitalized patients who did not undergo imaging compared to 42.2% of the patients who were referred to hospitalization and underwent imaging. The rate of hospitalization was also considerably higher in patients who had surgery in the ED than in patients who did not require surgery [71.1% vs. 12.9%; χ^2^ (1) = 198.24, *p* < 0.001]. Those with a longer surgical procedure were at the highest risk relative to those who had a shorter procedure (1.11 ± 1.20 vs. 0.02 ± 0.29 h, *p* < 0.001).

On logistic regression analysis (Table 4), the independent variables significantly predicted hospitalization [c^2^(15) = 194.48, *p* < 0.001], and together explained 61.9% of the total variance. The model had an acceptable fit to the data [c^2^(8) = 9.28, *p* = 0.32], classifying 97.2% of the total observations. The use of imaging in the ED decreased the odds of hospitalization 0.07-fold (*p* < 0.001), and every additional hour of surgery increased the odds of hospitalization 8.45-fold (*p* < 0.001).

Age at ED admission was not a significant predictor of the likelihood of hospitalization (OR = 1.02, 95%CI 0.99–1.06; *p* = 0.11).

Gender was also not a significant predictor (OR = 0.54, 95%CI 0.16–1.87; *p* = 0.33).

## 4. Discussion

Most previous studies of E-bike and P-scooter injuries have evaluated the gender and age of riders. The present study further investigated the impact of age on the severity of injuries in this patient group.

Although most riders involved in accidents have been found to be male [2,6,7,8,9,10], the present study showed that male gender was not associated with a significantly higher probability of being hospitalized for injuries. Age, however, was found to be a risk factor on univariate analysis, with patients who were hospitalized being significantly older than patients who were not (*p* < 0.001).

The worldwide increase in longevity in recent years and the consequent increase in the size of the older population have been accompanied by an increase in the number of visits to the ED [13,14]. In the USA, patients aged 75 years or more were found to be among the largest age groups accounting for general ED visits [15,16]. Others reported an association of older age with a higher incidence of hospital admission [14].

Our findings are supported by an earlier retrospective study of acute E-bike and P-scooter injuries based on the Singapore National Trauma Registry in 2016 [17]. The authors showed that among all personal mobility devices, E-bikes and P-scooters accounted for the most severe injuries (42.9% and 28.6%, respectively). Other important factors were site of injury, with injuries to the head, face and thorax being more severe, and older age.

Another retrospective study conducted in Vienna, Austria included patients admitted to three major trauma departments between May 2018 and September 2018 for electric- scooter-related injuries [4]. The results indicated that the Injury Severity Score increased with an increase in rider age and was significantly higher in patients aged ≥40 years than in younger patients (*p* = 0.011).

In the present study, on the one hand, findings for age on univariate analysis showed a relatively higher rate of admissions in the older age groups (Figure 3). On the other hand, the logistic regression model yielded an OR of 1.02 (*p* = 0.11), indicating that older age was not a predicting factor for hospital admission. Moreover, further analysis revealed that the difference in mean age between the hospitalized and nonhospitalized patients was only 6 years (31.08 ± 14.72 vs. 37.21 ± 14.34), and that the large majority (89%) of injured patients were less than 50 years old. Thus, the “older” population of E-bike and P-scooter riders in this and other relevant studies does not conform with the general population, where the truly old are aged 70 years or more. This may explain the discrepancy between the univariate and multivariate analyses [1,2,3,6,7,8,9,10,11]. This finding has important implications for healthcare administrators and insurance companies.

Mukhtar et al. [18] evaluated 192 patients with injuries associated with electric scooters. In 140 patients (72.9%), the injuries were identified on imaging evaluation, including radiographs (57.2%), computed tomography scans (42.3%), and computed tomography angiograms (0.4%). These results are in agreement with the present study, showing that plane radiographs followed by computed tomography are the most common imaging modalities used in this setting.

We found that when imaging was used in the diagnostic work-up, hospitalization was less likely [χ^2^ (1) = 111.45, *p* < 0.001], pointing to the importance of proper imaging in ED decision making. Imaging may decrease the load on the ED and admitting departments, while assuring that severe injury is not missed in the clinical examination so the patient can be safely discharged home.

Another interesting finding in our study was the association of surgery in the ED and of a longer operative time with an increased probability of hospitalization [χ^2^ (1) = 198.24, *p* < 0.001]. These results are in line with the well-known risk of surgical site infection in operated patients and the importance of adequate perioperative care [19]. A systematic review including 81 studies reported a statistically significant association between prolonged operative time and risk of surgical site infection [20]. The major limitation of the study may be the retrospective study design, and probably the absence of detailed questionnaire specified for electric vehicle injuries. Future studies are needed to assess the influence of other predictive factors that may increase the likelihood of hospitalization for injuries related to E-bikes and P-scooters.

## 5. Conclusions

Despite the widespread use of E-bikes and P-scooters, parameters impacting the risk of injuries and their severity have not been adequately investigated. In the present study, older age (>51 years) was not an independent predictor of referral for hospitalization from the ED of injured E-bike and P-scooter riders. Thus, the study hypothesis was refuted. This finding should alert hospital administrators and insurance companies to reconsider age as a reliable factor for predicting severe injury risk in this patent group. Future studies may be focused on different drivers’ characteristics as predictive factors in incidence and severity of injuries following electric bike and powered scooter rides.

## Figures and Tables

**Table 1:** Demographic and clinical characteristics.

Characteristics	Value
**Gender**	
**Male**	934 (75.7%)
**Female**	300 (24.3%)
**Age (year), mean ± SD**	31.52 ± 14.77
**<21 years**	284 (23.0%)
**21–30 years**	410 (33.2%)
**31–40 years**	285 (23.1%)
**41–50 years**	122 (9.9%)
**>51 years**	133 (10.8%)
**Vehicle**	
**E-bike**	980 (79.5%)
**P-scooter**	253 (20.5%)
**Imaging during ED work-up**	
**Plane radiographs**	1027 (83.23%)
**Computed tomography**	265 (21.47%)
**Ultrasound**	68 (5.51%)
**Enhanced computed tomography**	9 (0.73%)
**Magnetic resonance imaging**	0
**Other**	265 (21.47%)
**None**	100 (8.10%)
**Surgery**	
**No**	1021 (82.74%)
**Yes**	213 (17.26%)
**Duration (hr), mean ± SD**	0.10 ± 0.51
**Outcome after ED care**	
**Discharged home**	1218 (98.8%)
**Referred for hospitalization**	12 (1.0%)
**Died**	3 (0.2%)

Values are n(%) unless otherwise indicated.

**Table 2:** Study variables.

	Values	Normality Tests
**Sex**	Male/female	
**Age**		Non-normal, *p* < 0.001
**Vehicle**	Scooter/Electric bike	
**Imaging**	CT/enhanced CT/MRI/plane radiographs/US/none/ other	
**Number of admission days**		Non-normal, *p* < 0.001
**Operation**	None/Yes	
**Hours of operation**		Non-normal, *p* < 0.001
**Patient status at end of medical care**	Dead/released home/released to rehabilitation	

**Table 3:** Factors predicting the need for hospitalization.

Variable	Referred for Hospitalization	Discharged Home	*p* Value
**Gender**			0.12
**Male**	78 (86.7%)	856 (74.8%)	
**Female**	12 (13.3%)	288 (25.2%)	
**Age (year), mean ± SD**	37.21 ± 14.34	31.08 ± 14.72	<0.001
**Vehicle**			0.34
**E-bike**	68 (75.6%)	911 (79.8%)	
**P-scooter**	22 (24.4%)	231 (20.2%)	
**Imaging**			<0.001
**Yes**	38 (42.2%)	982 (85.9%)	
**No**	52 (57.8%)	161 (14.1%)	
**Surgery**			<0.001
**No**	26 (28.9%)	995 (87.1%)	
**Yes**	64 (71.1%)	148 (12.9%)	
**Duration of surgery (hr), mean ± SD**	1.11 ± 1.20	0.02 ± 0.29	<0.001
**Outcome**			<0.001
**Discharged home**	80 (88.9%)	1137 (99.6%)	
**Discharged for rehabilitation**	10 (11.1%)	2 (0.2%)	
**Died**	0	3 (0.3%)	

Values are n (%) unless otherwise indicated.

**Table 4:** Binary logistic regression coefficients predicting hospitalization.

	Odds Ratio	95% CI		*p* Value
		Lower	Upper	
**Gender (female)**	0.54	0.16	1.87	0.33
**Imaging**	0.07	0.03	0.19	<0.001
**Surgery**	1.97	0.64	6.05	0.24
**Hours of surgery**	8.45	3.16	22.57	<0.001
**Age**	1.02	0.99	1.06	0.11

## Data Availability

The datasets used and/or analyzed during the current study are available from the corresponding author on reasonable request.
